# Chromosome-Scale Genome Assembly for Soft-Stem Bulrush (*Schoenoplectus tabernaemontani*) Confirms a Clade-Specific Whole-Genome Duplication in Cyperaceae

**DOI:** 10.1093/gbe/evae141

**Published:** 2024-07-01

**Authors:** Yang Li, Yu Ning, Yan Chao Zheng, Xuan Yu Lou, Zhe Pan, Shu Bin Dong

**Affiliations:** Huzhou University, Huzhou, China; Wetland Research Center, Institute of Ecological Conservation and Restoration, Chinese Academy of Forestry, Beijing, China; Sichuan Zoige Wetland Ecosystem Research Station, Prefecture of Aba, China; East China Inventory and Planning Institute, Hangzhou, China; Zhejiang Wanli University, Ningbo, China; Sichuan Academy of Environmental Policy and Planning, Chengdu, China; College of Biological Sciences and Technology, Beijing Forestry University, Beijing, China

**Keywords:** *Schoenoplectus*, karyotype, Cyperaceae, holocentric chromosome, WGD

## Abstract

*Schoenoplectus tabernaemontani* (C. C. Gmelin) Palla is a typical macrophyte in diverse wetland ecosystems. This species holds great potential in decontamination applications and carbon sequestration. Previous studies have shown that this species may have experienced recent polyploidization. This would make *S. tabernaemontani* a unique model to study the processes and consequences of whole-genome duplications in the context of the well-documented holocentric chromosomes and dysploidy events in Cyperaceae. However, the inference was not completely solid because it lacked homology information that is essential to ascertain polyploidy. We present here the first chromosome-level genome assembly for *S. tabernaemontani*. By combining Oxford Nanopore Technologies (ONT) long reads and Illumina short reads, plus chromatin conformation via the Hi-C method, we assembled a genome spanning 507.96 Mb, with 99.43% of Hi-C data accurately mapped to the assembly. The assembly contig N50 value was 3.62 Mb. The overall BUSCO score was 94.40%. About 68.94% of the genome was comprised of repetitive elements. A total of 36,994 protein-coding genes were predicted and annotated. Long terminal repeat retrotransposons accounted for ∼26.99% of the genome, surpassing the content observed in most sequenced Cyperid genomes. Our well-supported haploid assembly comprised 21 pseudochromosomes, each harboring putative holocentric centromeres. Our findings corroborated a karyotype of 2*n* = 2*X* = 42. We also confirmed a recent whole-genome duplication occurring after the divergence between Schoenoplecteae and Bolboschoeneae. Our genome assembly expands the scope of sequenced genomes within the Cyperaceae family, encompassing the fifth genus. It also provides research resources on Cyperid evolution and wetland conservation.

**Significance**

The soft-stem bulrush (*Schoenoplectus tabernaemontani*) holds promise as a valuable wetland plant. The inadequacy of accessible genetic information impedes a comprehensive understanding of its ecological significance and evolutionary uniqueness. We present the inaugural chromosome-level genome assembly for *S. tabernaemontani*, characterized by competent quality and detailed annotation of protein-coding genes and repeated sequences. Our genome assembly substantiates a robust karyotype inference for the sequenced individual of *S. tabernaemontani* (2*n* = 2*X* = 42). We validate a clade-specific whole-genome duplication occurring after the divergence between Schoenoplecteae and Bolboschoeneae, contributing an example of duplication-driven evolution within the dysploidy-prevalent Cyperaceae family.

## Introduction

*Schoenoplectus tabernaemontani* (C. C. Gmelin) Palla, common name as soft-stem bulrush, is a flagship macrophyte in wetland ecosystems. It is a promising plant in decontamination applications. This species performs well in tolerating multiple organic pollutants, inorganic heavy metals, and nanoparticle (Zhang et al. 2009; Blanco 2018; Yan et al. 2022). However, debates exist about the biology and practical potential of *S. tabernaemontani*. For example, this species was reported to selectively retain arsenic and selenium in belowground tissues while conveying other heavy metals, such as lead, copper, and cadmium, to aboveground parts (Hammill et al. 2022). This selective strategy may lead to the accumulation of harmful elements among trophic levels. The notorious immunity of *Schoenoplectus* plants to herbicides also has negative effects on crop production (Scarabel et al. 2009). Nevertheless, *Schoenoplectus* plants have critical ecological significance. They typically grow fast and yield high biomass. Previous studies have shown that they are competent nontimber materials in construction practices, offering an alternative way to limit carbon emissions (Hidalgo-Cordero and García-Navarro 2018). Research on coastal wetlands also highlighted the heritable variations in the biomass allocation strategy of *Schoenoplectus americanus* and its relations with estuary carbon sequestration and soil surface accretion (Blum et al. 2021; Vahsen et al. 2023). However, a high-quality reference genome for *S. tabernaemontani* is still lacking, hindering further insights into the biological mechanisms of this promising plant.

*Schoenoplectus tabernaemontani* belongs to the species-rich sedge family (Cyperaceae). The prevalence of holocentric chromosomes confers evolutionary uniqueness to Cyperaceae species (Escudero et al. 2012, 2016; Hofstatter et al. 2022). The pervasive distribution of centromeres along the entire chromosome facilitates tolerance to breakages of chromosomes, which may prompt speciation through dysploidy instead of polyploidy (Lucek et al. 2022). For example, polyploidy occurrence is strikingly low in the *Carex* genus, despite the high volume of species diversity (∼2,000) and an exceptional chromosome number variation (2*n* = 10 to 132) (Márquez-Corro et al. 2021). However, it may not hold for other Cyperid species, as the chromosome number could evolve at heterogeneous rates along different clades (Márquez-Corro et al. 2019; Shafir et al. 2023). Notably, previous studies have provided some clues for polyploidization in the *Schoenoplectus* genus. Yano and Hoshino (2005) have examined 13 *Schoenoplectus* species, revealing a set of varied chromosome numbers, but individual chromosome sizes nearly hold constant, indicating a larger chance of polyploidy than dysploidy. The first record of polyploid intraindividual variation has also been found in *Schoenoplectus acutus* (Tena-Flores et al. 2014). Nevertheless, most of the evidence comes from chromosome counting, lacking homology information that is critical in inferring polyploidy events, especially autopolyploidy (Spoelhof et al. 2017). Thus, we present here the first chromosome-scale genome assembly of *S. tabernaemontani*, expanding the scale of the Cyperaceae reference genomes to the fifth genus. We aim to provide a valuable genetic resource for research on Cyperaceae evolution and wetland conservation.

## Results and Discussion

### Competence of the Genome Assembly

In total, we acquired 55.48 Gb (∼112×) of Oxford Nanopore Technologies (ONT) long-read data for preliminary assembly, 46.50 Gb (∼94×) of Illumina short-read data for genome profiling and back-mapping check, 45.04 Gb (∼91×) of Hi-C (all-vs.-all chromosome conformation capture) data for pseudochromosome construction, and 14.08 Gb (∼28×) of RNA-seq data for gene prediction. The average Q30 value for our short-read data was 92.76. The mean Q value for ONT data was 11.50. (supplementary fig. S1 and table S1, Supplementary Material online). Results of genome profiling showed the sequenced genome was moderately complex (∼1.3% heterozygosity). The inferred genome size was about 513 Mb, with repetitive content of ∼48.40% and GC content of ∼33.26% (supplementary fig. S2, Supplementary Material online). The estimated genome size is consistent with all four records in the comprehensive research by Elliot et al. (2022) about genome and chromosome evolution in Cyperid species, which provide essential guidance for our further assembling.

**Fig. 1.**
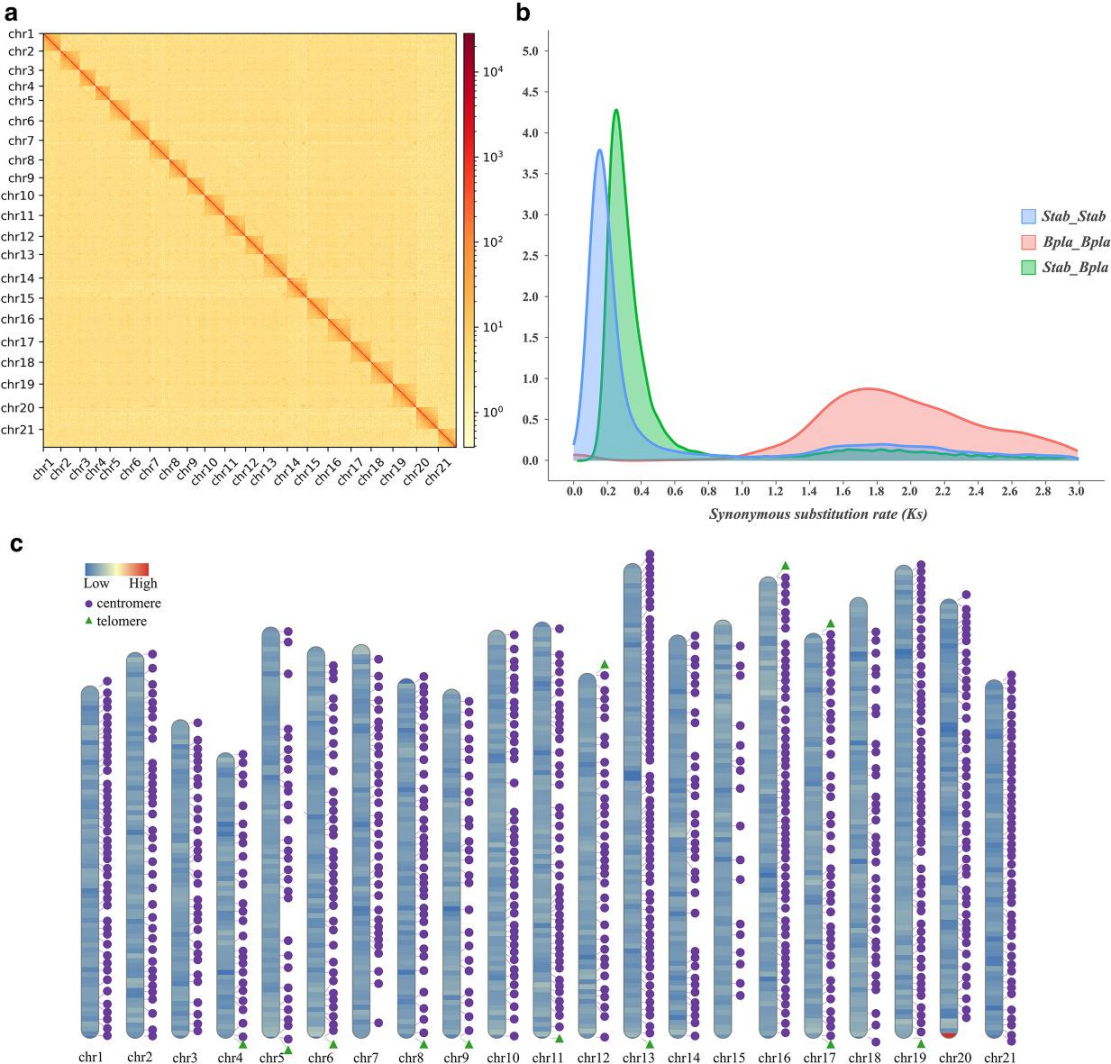
Summary of the *S. tabernaemontani* genome assembly. a) Heatmap of Hi-C (all-vs.-all chromosome conformation capture) interactions within *S. tabernaemontani* pseudochromosomes. Gradients in the scale bar indicate the frequencies of Hi-C links alter from low to high. b) Density plot of the synonymous substitution rate (*K_s_*) showing the different evolution modes for *B. planiculmis* (*Bpla*) and *S. tabernaemontani* (*Stab*). A clear peak is detected in the *S. tabernaemontani* genome subsequent to its divergence from *B. planiculmis*. c) Chromosome ideograms showing the karyotype of the assembled genome. Length of ideograms is proportional to chromosome size. Filling color scales with gene densities (300-kb window size). The putative telomeres are indicated with green triangle. The detected centromeres (purple circle) manifest pervasive distribution.

Using ONT data, we assembled a preliminary genome (supplementary table S2, Supplementary Material online). Then, we incorporated high-quality Hi-C data (supplementary table S3, Supplementary Material online) and polished this genome to chromosome level (Table 1). We successfully detected the association between most contigs. These contigs were then clustered into pseudomolecules. Eventually, we constructed a haploid assembly of 21 pseudochromosomes (Fig. 1a; supplementary table S4, Supplementary Material online). Up to 99.43% of the total bases were mapped into these pseudochromosomes. Our final assembly showed that *S. tabernaemontani* has a 1C genome size of 507.96 Mb (including both the well-mapped and unmapped bases). The contig N50 value is 3.62 Mb. The scaffold N50 value is 24.61 Mb. Detailed information on the assembly was listed in Table 1. We also provided a Circos graph (supplementary fig. S3, Supplementary Material online) that shows the gene density, GC content, transposable elements (TEs), and intragenome collinearity relations.

**Table 1:** Statistics for the *S. tabernaemontani* genome assembly and BUSCO scores. “Anchored rate” refers to the proportion of bases that are well mapped into pseudochromosomes. Those unmapped bases are also included in the final assembly. “Size range” delimits the minimum and maximum size of pseudochromosomes

Type	Statistics
***Sequence***	
Assembly size (bp)	507,964,631
GC content (%)	33.32
Number of scaffolds	57
Scaffold N50 size (bp)	24,610,677
Scaffold N90 size (bp)	21,215,737
Number of contigs	249
Contig N50 size (bp)	3,615,529
Contig N90 size (bp)	1,429,834
***Pseudochromosome***	
Number	21
Anchored rate (%)	99.43
Size range (M)	17.35 to 28.85
***BUSCO score***	
Complete BUSCOs (%)	94.40
Complete and single-copy BUSCOs (%)	70.30
Complete and duplicated BUSCOs (%)	24.10
Fragmented BUSCOs (%)	1.90
Missing BUSCOs (%)	3.70
Total groups searched	1,614

The quality of our genome assembly was supported by the following evidence: (i) The construction of pseudochromosomes was reliable. The mapping rate of Hi-C data (99.43%) was higher than formerly published genomes *Bolboschoenus planiculmis* (93.34%) (Ning et al. 2024) and *Carex littledalei* (96.28%) (Can et al. 2020). The results of our chromosome-staining experiment also supported the haploid chromosome number of 21 (supplementary fig. S4, Supplementary Material online). This value was also consistent with previous studies (2*n* = 42) (Elliot et al. 2022); (ii) the complete BUSCO score (94.40%) was at a comparable level to those recently reported for four Cyperid genomes (Planta et al. 2022). The back-mapping scores were good. About 98.33% Illumina short reads got projection in the genome assembly, and ∼96.74% of the whole genome was covered through back mapping; (iii) successful detection of telomeres and centromeres consolidated the high quality. Although highly repetitive in base content, centromeres and telomeres are vital components in gene regulation and cell biology (Lin et al. 2023). Both elements act as key criteria in the evaluation of telomere-to-telomere genome assembly. In our case, telomeres were detected in 11 pseudochromosomes (∼52.38% of the total), with Chr17 showing signals at both ends (Fig. 1c). Notably, our assembly supported a diffused distribution of centromeres along each chromosome, indicating that *S. tabernaemontani* may host holocentric chromosomes. Holocentricity has long been recognized as a critical and flexible trait in the diversification of Cyperaceae species (Escudero et al. 2016, 2012; Hofstatter et al. 2022). Our new assembly provides data resources that may benefit future research to fully ascertain the specific mechanisms of holocentricity in *S. tabernaemontani*.

### Repetitive Elements and Gene Annotation

Repetitive elements constituted about 68.94% (∼350.19 Mb) of the *S. tabernaemontani* genome. Approximately 55.33% of the genome was composed of TEs. Tandem repeats consisted of ∼13.61% of the genome (see details in supplementary tables S5 and S6, Supplementary Material online). Based on the repeat-masked genome, we predicted protein-coding genes through a combination of three methods: ab initio, homology, and transcriptome-based prediction. In total, 36,994 protein-coding genes were identified in the *S. tabernaemontani* genome. Detailed information about gene prediction and BUSCO scores is presented in supplementary table S7, Supplementary Material online. The complete and duplicated type notably scored 22.00%, suggesting a potential large-scale duplication event. Approximately 91.76% of all the predicted genes got annotated at canonical databases (Pfam, EggNOG, Swiss-Prot, KEGG, NR, KOG, GO, and TrEMBL; see details in supplementary table S7, Supplementary Material online). We also established a computationally predicted noncoding RNA library, consisting of 550 rRNAs, 625 tRNAs, 200 miRNAs, and 464 snRNAs (supplementary table S8, Supplementary Material online).

Notably, the proportion of long terminal repeat retrotransposons (LTR-RTs) in *S. tabernaemontani* genome ranked high in all the available Cyperaceae genome assemblies (supplementary table S9, Supplementary Material online). Previous studies have shown that chromosomes originated from fusion (leading to large chromosome) may possess higher amounts of repetitive DNA, whereas fission (leading to small chromosome) may favor effective purge of repeat contents (Bureš and Zedek 2014; Veleba et al. 2016). Our genome assembly (26.99% LTR-RTs, mean chromosome size ∼24.19 Mb) provides preliminary clues for putative fusion events in the genome of *S. tabernaemontani.*

### WGD and Clade-Specific Evolution Mode

Our genome assembly confirmed a clade-specific whole-genome duplication (WGD) event. The synonymous substitution rate (*K_s_*) distribution clearly showed a burst after the divergence between *S. tabernaemontani* and *B. planiculmis* (Fig. 1b). It is also supported by the apparently higher amount of complete and duplicated BUSCOs (22.00%) compared with other species lacking genetic duplication, e.g. *Cyperus esculentus* (1.50%) (Zhao et al. 2023) and *B. planiculmis* (1.49%) (Ning et al. 2024). The strongest evidence came from the considerable amounts of collinear blocks within the *S. tabernaemontani* genome (supplementary fig. S5, Supplementary Material online). Previous studies have shown that intragenome collinear segments amend the possible deceiving effect of *K_s_* plot, especially in inferring WGD events among recent divergent lineages (Zwaenepoel et al. 2019). Thus, the clade-specific WGD in *S. tabernaemontani* is highly possible. The prevalence of dysploidy evolution is well documented in some lineages in Cyperaceae. Our result exhibited a contrary instance. However, our result did not support polyploidy in *S. tabernaemontani*, as the genome profiling shows the karyotype to be 2*n* = 2*X* = 42 (supplementary figs. S2 and S4, Supplementary Material online). Furthermore, this result may provide valuable information in the transitions of evolution mode among closely related clades. Márquez-Corro et al (2019) have highlighted that, in the Fuireneae–Abildgaardieae–Eleocharideae–Cypereae clade, Cypereae showed a strikingly high rate of dysploidy events compared with the remarkably low rate of chromosome evolution in the rest lineages (*Schoenoplectus* included). Our inference of WGD offered a possible explanation other than chromosome number variation.

## Materials and Methods

### Collection and Preparation of Plant Materials

The sequenced samples were taken from a healthy individual of *S. tabernaemontani* at the Yongding wetland (39.887°N, 116.177°E). The sampled individual was well maintained in its original habitat for long-term research purpose. We selected vigorous leaves and treated them with caution to avoid exogenous contamination. All the field samples were swiftly transferred to lab environment and stored at −80 °C.

### Genome Sequencing

We followed the cetyltrimethylammonium bromide method to extract genomic DNA. We checked the quality of DNA extraction through agarose gel electrophoresis. The SQKLSK109 ligation kit was used to generate ONT libraries. Primed R9.4 Spot-On Flow Cells were prepared following standard protocols to settle the purified libraries. We chose the PromethION platform to execute the sequencing. The raw data were treated using the Oxford Nanopore GUPPY software (v.0.3.0). Technical details could be found at https://github.com/nanoporetech. For Illumina short-read sequencing, pair-end libraries were constructed using the Nextera DNA Flex Library Prep Kit (Illumina, San Diego, CA, USA) and sequenced on the NovaSeq 6000 platform. We chose SOAPnuke (v.2.1.4) tool to clean and filter the raw reads (https://github.com/BGI-flexlab/SOAPnuke).

### Transcriptome Sequencing

For gene prediction, total RNA was extracted and sequenced from four independent tissue samples (stem, tuber, spikelet, and root). The extraction of RNA was established following the manufacturer's instructions on RNA prep Pure Plant Plus Kit (Tiangen Biotech [Beijing] Co., Ltd., China). Then, the samples were pooled and sequenced on the Illumina NovaSeq 6000 platform. The library type was paired-end. The insertion size was about 350 bp on average. The generation of library followed the standard protocols of Illumina.

### Genome Profiling and Draft Assembly

Genome profiling was realized using Genome Scope (v.2.0) (Ranallo-Benavidez et al. 2020) and Jellyfish (v.2.1.4) (Marçais and Kingsford 2011). The primary assembly was acquired using the NextDenovo pipeline (https://github.com/Nextomics/NextDenovo). Double rounds of error check of the primary assembly were performed using both the ONT data and the Illumina data. Heterozygous sequences were removed from the error-checking assembly using Purge_haplotigs pipeline (v.1.0.4) (Roach et al. 2018) to decrease ambiguities.

For Hi-C library construction, we followed a previously published protocol involving HindIII enzymatic digestion (Xie et al. 2015). The clean Hi-C data were then aligned with the draft assembly using Burrows–Wheeler Aligner (v.0.7.17) (Li and Durbin 2009). Only read pairs that were uniquely aligned were deemed valid-interaction reads. HiCUP (v.0.8.0) (Wingett et al. 2015) was used to screen and filter out read pairs. We clustered the contigs of the draft assembly into several groups (pseudochromosomes) using ALLHiC (v.0.9.8) (Wang and Zhang 2022). The orientation and ordination of contigs were further improved using 3D-DNA (v.180922) (Dudchenko et al. 2017) and Juciebox (v.1.11.08) (Durand et al. 2016).

### Detection of Repetitive Elements

A de novo repeat library was acquired using RepeatModeler (v.2.0.1) (Flynn et al. 2020). We utilized a pipeline incorporating LTR_finder, LTR_harvester, and LTR_retriever to identify high-quality LTRs (Ou and Jiang 2017). RepeatMasker (v.4.15) and RepBase (v.20181026) were jointly used to finalize the repeat library. We utilized TRF (v.4.1.0) (Benson 1999) and MISA (v.2.1) (Beier et al. 2017) to annotate tandem repeats. Python scripts of quarTeT (Lin et al 2023) were used to detect potential centromeres and telomeres. Visualization was established using RIdeogram (v.0.2.2) (Hao et al. 2020).

### Gene Prediction and Annotation

Based on the repeat-masked genome, Augustus (v 3.5.0) was utilized to generate de novo gene models (Stanke et al. 2008). The homology-based inference was achieved by using five well-annotated species as references (*Arabidopsis thaliana*, *Oryza sativa*, *Triticum aestivum*, *Rhynchospora breviuscula*, and *B. planiculmis*). TransDecoder (v.5.7.1) (https://github.com/TransDecoder/TransDecoder) was applied to parse the transcripts. Finally, these three types of evidence were integrated and reconciled using Maker (v.3.01) pipeline to obtain ultimate gene prediction results (https://github.com/Yandell-Lab/maker?tab). For noncoding RNA, we used tRNAscan-SE (v.1.3.1) (Lowe and Eddy 1997) to detect tRNA with eukaryote parameters. We used RNAmmer (v.1.2) to identify rRNA genes (https://services.healthtech.dtu.dk/services/RNAmmer-1.2/). We used a combination of Infernal (v.1.1.4) (Nawrocki and Eddy 2013) and Rfam (v.14.9) (Kalvari et al. 2021) to determine the miRNA, snoRNA, and snRNA in this genome. Both the Infernal and the Rfam incorporate covariance models. These models consider RNA secondary structure and primary sequence simultaneously, which greatly improves the scope of potential candidates (Kalvari et al. 2018).

### Detection of Intragenome Synteny and WGD

We utilized the WGDI toolkit (Sun et al. 2022) to reveal the intragenomic synteny among pseudochromosomes and the potential WGD events. By implementing a hierarchical algorithm, WGDI has been shown to have high sensitivity and accuracy in collinearity detection. We applied the built-in functions of “-d”, “-icl”, “-ks”, “-bi”, and “-bk” to generate our inferences. Finally, we got an ideogram of pseudochromosomes to intuitively represent the multidimensional genomic information. The visualization of synonymous substitution (*K_s_*) burst was accomplished using ggplot2 (https://github.com/tidyverse/ggplot2).

## Supplementary Material

evae141_Supplementary_Data

## Data Availability

The presented genome is deposited in the NCBI (Genome assembly ID CAF_SchTab_1.0, Bioproject ID PRJNA1055192, and Biosample ID SAMN38984562). The raw reads used to generate this genome assembly are stored at Sequence Read Archive (SRA) (ONT long-reads: SRR27340954; Illumina short reads: SRR27340955). The complete set of genome annotation files in gff3 format, including coding sequences, ncRNA sequences, and repeat sequences, are shared at https://figshare.com/articles/dataset/Annotation_files_for_the_chromosome-level_genome_assembly_of_soft-stem_bulrush_i_i_i_Schoenoplectus_tabernaemontani_i_/25367605.
