# Supplementary material for: Chromosome-Scale Genome Assembly for Soft-Stem Bulrush (Schoenoplectus tabernaemontani) Confirms a Clade-Specific Whole-Genome Duplication in Cyperaceae
Source: Genome Biol Evol. 2024 Jul 1;16(7):evae141. doi: 10.1093/gbe/evae141 (PMC11251425; doi:10.1093/gbe/evae141)
Supplement: evae141_Supplementary_Data [file evae141_supplementary_data.docx]

|  |
| --- |

**Supplementary materials for “Chromosome-scale genome assembly for soft-stem bulrush (*Schoenoplectus tabernaemontani*) confirms a clade-specific whole genome duplication in Cyperaceae”**

LI, Yang^1^; NING, Yu^2,3,*^; ZHENG, Yan Chao^4^, LOU, Xuan Yu^5^; PAN, Zhe^6^; DONG, Shu Bin^7^;

*1: Huzhou University, Huzhou, China;*

*2: Wetland Research Center, Institute of Ecological Conservation and Restoration, Chinese Academy of Forestry, Beijing, China*

*3: Sichuan Zoige Wetland Ecosystem Research Station,Prefecture of Aba,China*

*4: East China Inventory and Planning Institute, Hangzhou, China*

*5: Zhejiang Wanli University, Ningbo, China*

*6: Sichuan Academy of Environmental policy and planning, Chengdu, China*

*7: College of Biological Sciences and Technology, Beijing Forestry University, Beijing, China*

^*^Corresponding author. Email: ningyucaf@caf.ac.cn

Address: 2#, Xiangshan Road, Haidian District, Beijing, China.

Phone &Fax: +86 01062824182


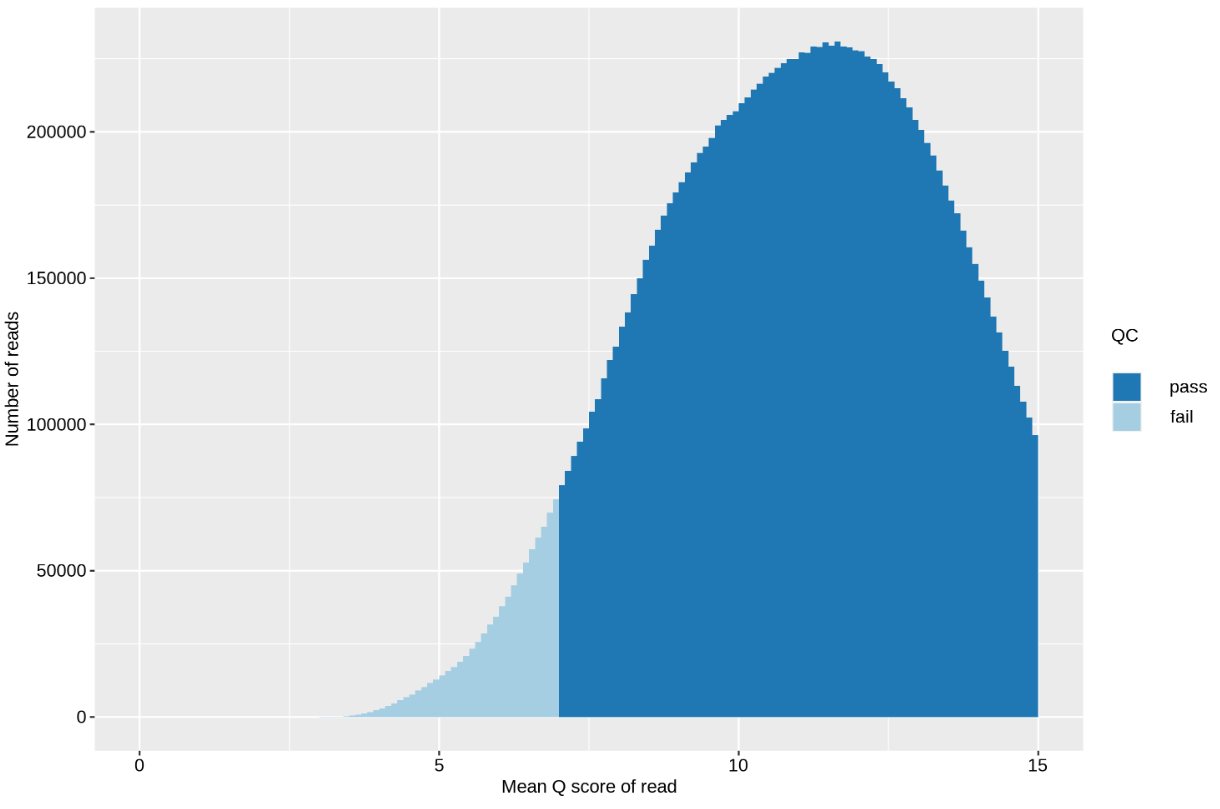


**Supplementary Figure S1. The distribution of quality scores across all reads generated using Oxford Nanopore Technologies (ONT) sequencing.** The bottom X-axis represents the mean Q score of reads. The left Y-axis shows the number of reads. The dark blue area represents the distribution of those qualified reads while the light blue color indicates those unqualified reads. The threshold of pass is Q7.


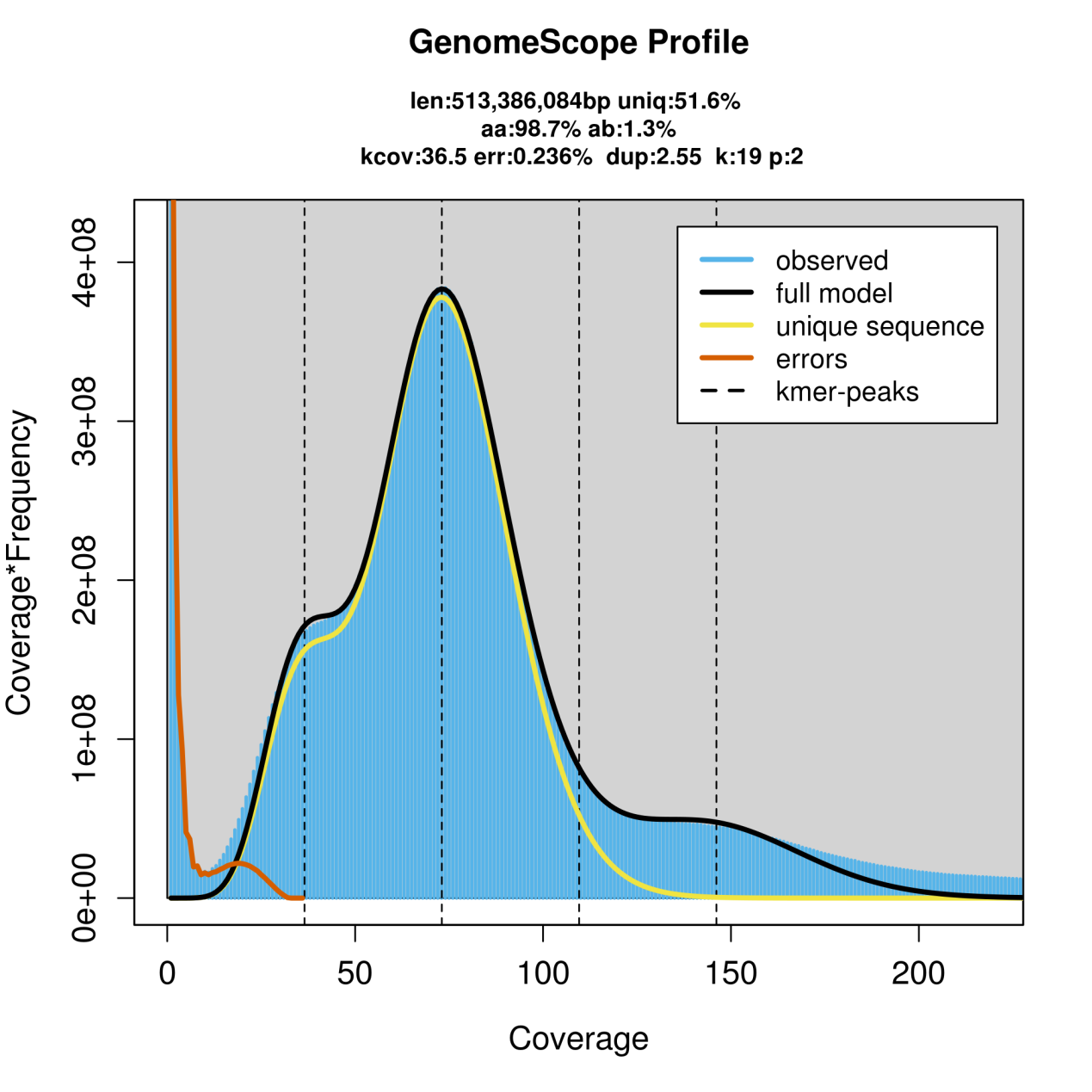


**Supplementary Figure S2. The *K*-mer analysis used to evaluate** ***Schoenoplectus tabernaemontani* genome.** The frequency distributions of 19-mers were shown.The genome size of *S.tabernaemontani* was estimated to be ~513 Mb, with approximate repetitive content of 48.40%, GC content of 33.26% and heterozygosity of 1.30%. The putative ploidy level is 2X.


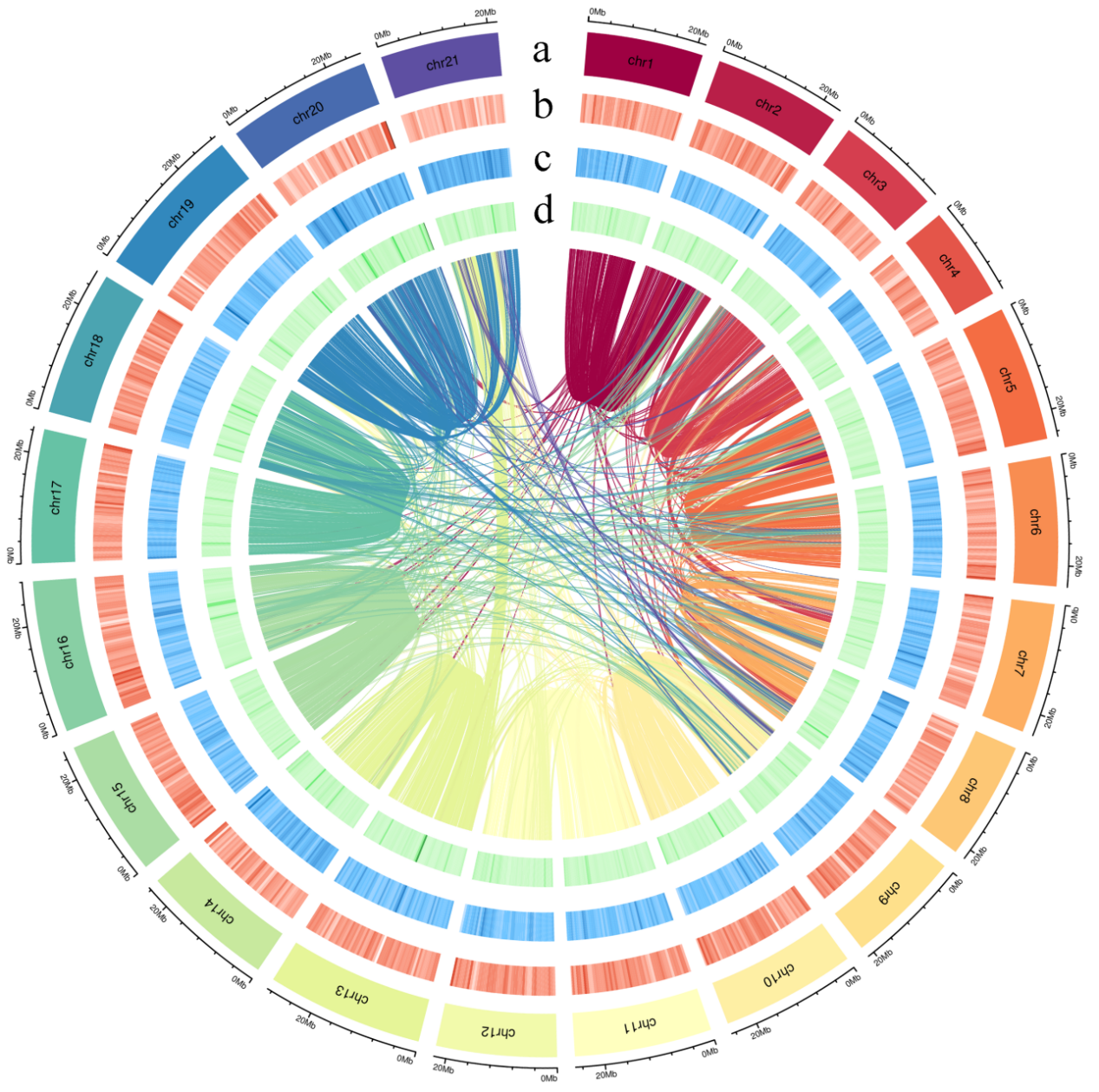


**Supplementary Figure S3. Circos plot showing the distribution of genomic features of *S. tabernaemontani*** The four circular tiers (a–d) represent chromosome ideograms, gene density, transposable-element density, and GC content, respectively. Central colored lines indicate putative homology among linked sections. The colors of these links are arbitrary and for visual purposes only.


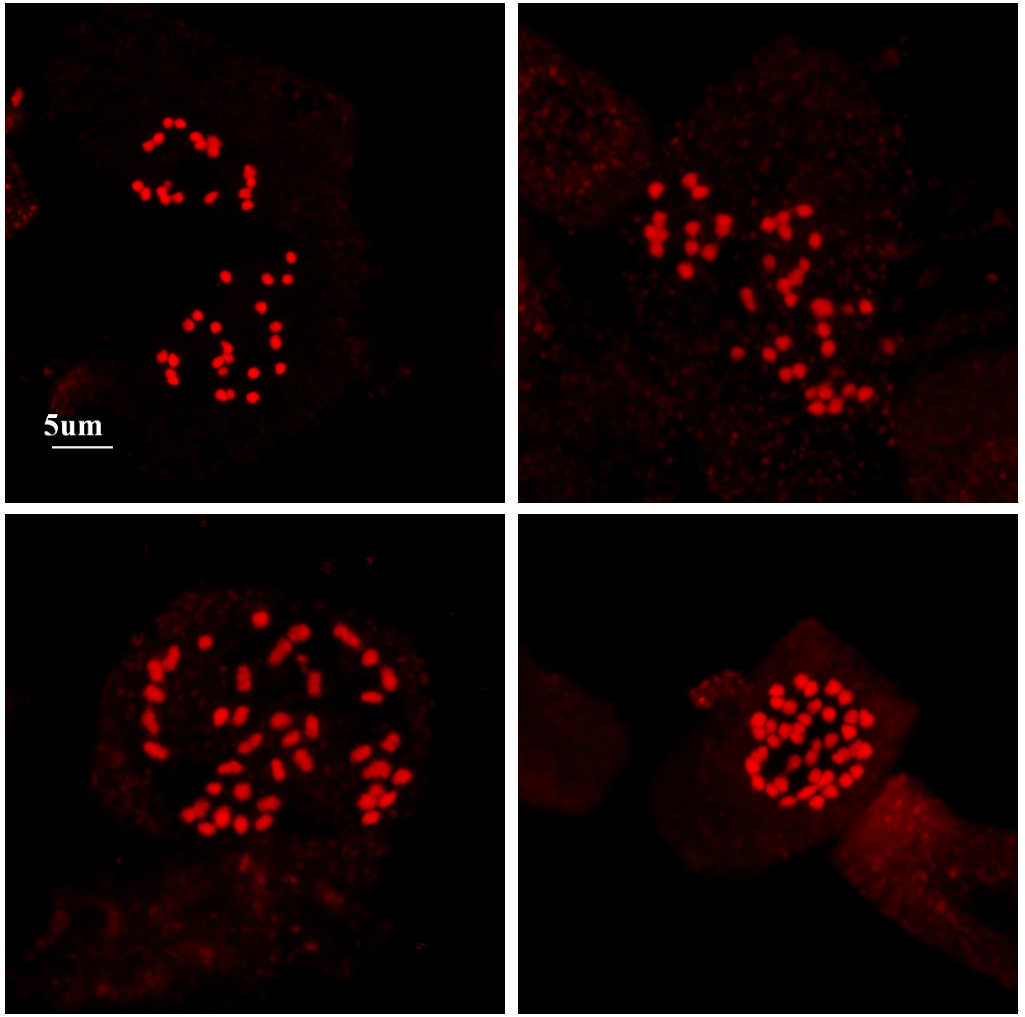


**Supplementary Figure S4. The result of DAPI staining experiment (4’,6-diamidino-2-phenylindole) on *S. tabernaemontani*.** These results show a well-supported karyotype of 2n = 2x =42. They also consolidate our genome assembly using Hi-C techonology which confirm a haploid of 21 pseudochromosomes.

| **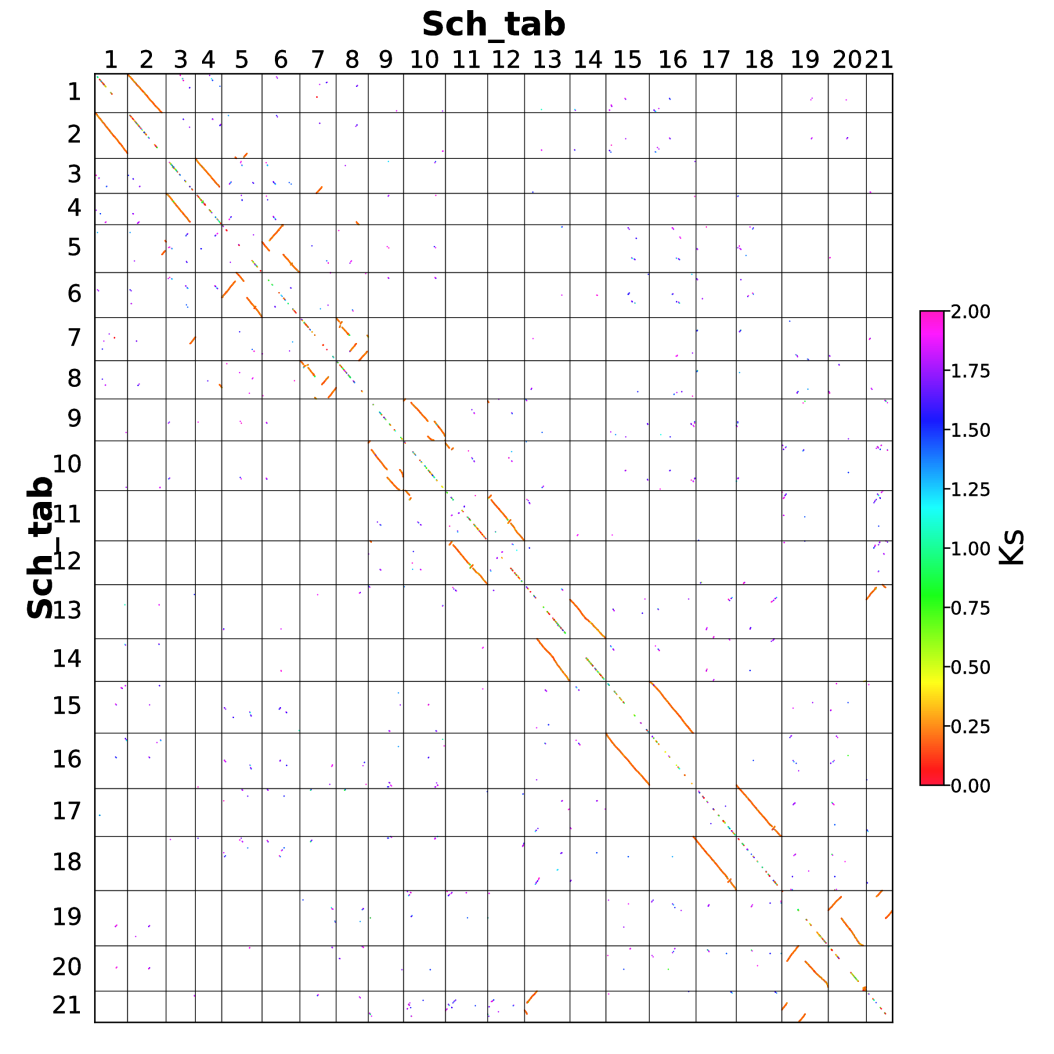**  **a** |
| --- |
| 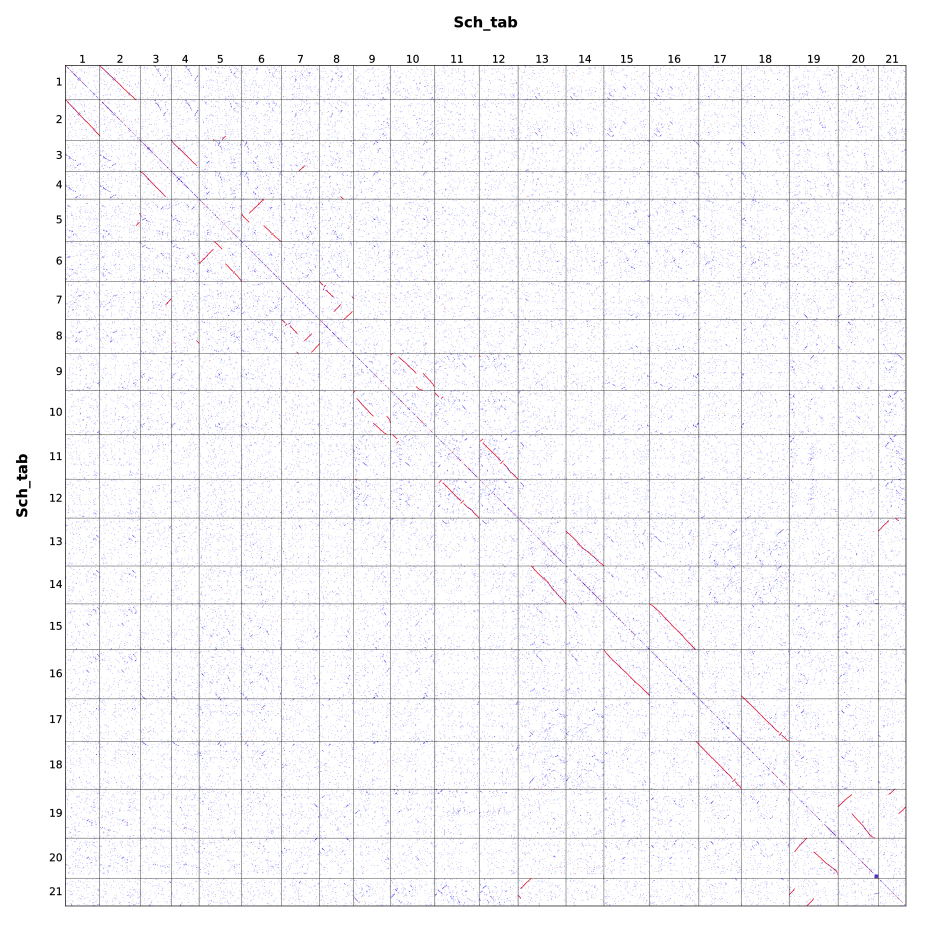  **Supplementary Figure S5. Filtered collinearity dot plot of gene pairs of *S. tabernaemontani* (a) and original unfiltered dot plot (b)**  Ks values are  indicated by color  gradients. Convincing  synteny blocks have been detected. These  blocks support recent  WGD event.  **b** |

**Supplementary Table S1. Summaries of data volume and quality**

| **Purpose** | **Library type** | **Platform** | **Total Data (Gb)** | **Reads Number** | **Quality** |
| --- | --- | --- | --- | --- | --- |
| preliminary assemble | ONT | PromethION P48 | 55.48 | 15,438,973 | 11.50 (mean Q) |
| Hi-C | Illumina | NovaSeq 6000 | 45.04 | 303,295,876 | 92.94 (Q30) |
| genome profiling | Illumina | NovaSeq 6000 | 46.50 | 312,817,034 | 92.96 (Q30) |
| *de novo* gene prediction | Illumina | NovaSeq 6000 | 14.08 | 46,937,332 | 92.38 (Q30) |

**Supplementary Table S2.** **Statistics of the preliminary assembly
of *S. tabernaemontani***

| **Species** | ***Schoenoplectus tabernaemontani*** | |
| --- | --- | --- |
| *Sequence* | |  |
| Assembly size (bp) | | 507,945,431 |
| GC content (%) | | 33.32 |
| Number of contigs | | 201 |
| Contig N90 size (bp) | | 1,472,098 |
| Contig N50 size (bp) | | 4,035,172 |
|  | |  |
| *BUSCO score* | |  |
| Complete BUSCOs (%) | | 94.40 |
| Complete & single-copy BUSCOs (%) | | 70.30 |
| Complete & duplicated BUSCOs (%) | | 24.10 |
| Fragmented BUSCOs (%) | | 1.90 |
| Missing BUSCOs (%) | | 3.70 |
| Total groups searched | | 1614 |

**Supplementary Table S3 Assessment of Hi-C library quality based on ratio of read pairs with valid interaction**

| **Type** | **Number** | **Ratio(%)** |
| --- | --- | --- |
| ***Total Unique Mapped*** | 59,100,701 |  |
| ***Valid Interaction*** | 53,962,845 | 91.31 |
| ***Invalid Interaction*** |  |  |
| Dangling End Pairs | 689,793 | 1.17 |
| Re-ligation Pairs | 1,583,555 | 2.68 |
| Self-cycle Pairs | 161,337 | 0.27 |
| Dumped Pairs | 2,703,171 | 4.57 |

**Supplementary Table S4** **Summary of *S. tabernaemontani* pseudochromosomes**

| **ChromosomeID** | **Length (bp)** | **Number of contigs** | **Number of genes** |
| --- | --- | --- | --- |
| Chr01 | 21,413,089 | 7 | 1510 |
| Chr02 | 23,450,444 | 11 | 1787 |
| Chr03 | 19,357,172 | 7 | 1357 |
| Chr04 | 17,351,366 | 10 | 1221 |
| Chr05 | 24,990,793 | 12 | 1865 |
| Chr06 | 23,795,600 | 8 | 1750 |
| Chr07 | 23,928,449 | 18 | 1678 |
| Chr08 | 21,856,963 | 12 | 1489 |
| Chr09 | 21,215,737 | 6 | 1635 |
| Chr10 | 24,801,447 | 11 | 1935 |
| Chr11 | 25,293,515 | 7 | 1955 |
| Chr12 | 22,179,320 | 4 | 1706 |
| Chr13 | 28,851,249 | 14 | 2106 |
| Chr14 | 24,505,306 | 14 | 1664 |
| Chr15 | 25,418,176 | 11 | 2010 |
| Chr16 | 28,045,748 | 8 | 2161 |
| Chr17 | 24,610,677 | 7 | 1865 |
| Chr18 | 26,793,420 | 8 | 2106 |
| Chr19 | 28,750,864 | 7 | 2150 |
| Chr20 | 26,695,277 | 21 | 1764 |
| Chr21 | 21,779,538 | 10 | 1215 |
|  |  |  |  |
| Average(±SD) | 24,051,626 (±2,901,666.67) | 10.14 (±4.00) | 1758.52 (±278.32) |
| Total | 505,084,150 | 213 | 36929 |

**Supplementary Table S5** **Classification of transposable elements in the *S. tabernaemontani* genome.**

| **Type** | **Length(bp)** | **Rate(%)** |
| --- | --- | --- |
| ***class Ⅰ Retroelement*** | **161954567** | **31.88** |
| LINE | 23642409 | 4.65 |
| **LTR** | **137081789** | **26.99** |
| Caulimovirus | 28253 | 0.01 |
| Copia | 38403463 | 7.56 |
| DIRS | 49250 | 0.01 |
| ERV1 | 106761 | 0.02 |
| ERVK | 108160 | 0.02 |
| Gypsy | 39976090 | 7.87 |
| Pao | 138844 | 0.03 |
| Unknown | 58270968 | 11.47 |
| SINE | 1230369 | 0.24 |
|  |  |  |
| ***class Ⅱ DNA transposon*** | **90677325** | **17.86** |
| CMC | 2973690 | 0.58 |
| Crypton | 112941 | 0.02 |
| DNA | 333428 | 0.07 |
| Ginger | 57351 | 0.01 |
| hAT | 7398010 | 1.46 |
| Helitron | 782764 | 0.15 |
| Kolobok | 36785 | 0.01 |
| Maverick | 127261 | 0.03 |
| Merlin | 27064 | 0.01 |
| MULE | 7413734 | 1.46 |
| P | 111581 | 0.02 |
| PIF | 268069 | 0.05 |
| TcMar | 385744 | 0.08 |
| unknown | 69447926 | 13.67 |
| Zisupton | 1200977 | 0.24 |
|  |  |  |
| ***Unknown*** | **28376132** | **5.59** |
|  |  |  |
| **TOTAL** | **281008024** | **55.33** |

**Supplementary Table S6 Classification of tandem repeats in the *S. tabernaemontani* genome.**

| **Type** | **Number** | **Length** | **Rate(%)** |
| --- | --- | --- | --- |
| Microsatellite (1-9 bp units) | 317,382 | 10,419,270 | 2.051 |
| Minisatellite (10-99 bp units) | 203,135 | 24,345,616 | 4.793 |
| Satellite (>=100 bp units) | 26,737 | 31,601,094 | 6.221 |
|  |  |  |  |
| Total | 547,254 | 51,212,027 | 13.605 |

**Supplementary Table S7.** **Summary of the gene prediction and annotation results**

| **Type** | **Statistics** |
| --- | --- |
| *Gene Prediction* |  |
| Number of predicted genes | 36,994 |
| Mean mRNA length (bp) | 3,889.41 |
| Mean CDS length (bp) | 1,076.35 |
| Total number of exon | 198,604 |
| Mean exon length (bp) | 273.47 |
| Total number of intron | 161,610 |
| Mean intron length (bp) | 551.5 |
|  |  |
| *Gene Annotation* |  |
| NR Annotated Percent (%) | 87.36 |
| Uniprot Annotated Percent (%) | 87.32 |
| GO Annotated Percent (%) | 24.54 |
| KEGG Annotated Percent (%) | 35.66 |
| Interpro Annotated Percent (%) | 89.37 |
| Pfam Annotated Percent (%) | 65.74 |
| Annotated in ≥ 1 Database Percent (%) | 91.76 |
| *Gene prediction BUSCO* |  |
| Complete BUSCOs (%) | 95.30 |
| Complete & single-copy  BUSCOs (%) | 73.30 |
| Complete & duplicated  BUSCOs (%) | 22.00 |
| Fragmented BUSCOs (%) | 0.50 |
| Missing BUSCOs (%) | 4.20 |
| Total groups searched | 1614 |

**Supplementary Table S8. Summary of the non-coding RNA (ncRNA) prediction and annotation results**

| Type | Copy | Average length(bp) | Total length(bp) | Rate(%) |
| --- | --- | --- | --- | --- |
| ***miRNA*** | **200** | **134** | **26,764** | **0.0053** |
| ***tRNA*** | **625** | **76** | **47,691** | **0.0094** |
| ***rRNA*** | **550** | **356** | **195,529** | **0.0385** |
| 18S | 27 | 1747 | 47,168 | 0.0093 |
| 28S | 23 | 3868 | 88,973 | 0.0175 |
| 5.8S | 178 | 121 | 21,484 | 0.0042 |
| 5S | 322 | 118 | 37,904 | 0.0075 |
| **snRNA** | **464** | **118** | **54,856** | **0.0108** |
| CD-box | 313 | 105 | 33,015 | 0.0065 |
| HACA-box | 55 | 131 | 7,195 | 0.0014 |
| splicing | 96 | 153 | 14,646 | 0.0029 |
|  |  |  |  |  |
| **TOTAL** | **1839** |  | **324840** | **0.064** |

**Supplementary Table S9 Comparison of transposable elements(TEs) among available Cyperid genomes**

| Organism_name | Genome_size(Mb) | GC_contents(%) | TE_LTRrts(%) | TE_Class I(%) | TE_Class II(%) | Total_TE(%) | literature_source |
| --- | --- | --- | --- | --- | --- | --- | --- |
| *Carex littledalei* | 373.85 | 35.44 | 27.87 | 33.39 | 18.53 | 55.34 | <https://doi.org/10.1038/s41597-020-0518-3> |
| ***Schonoplectus tabernaemontani*** | **507.96** | **33.32** | **26.99** | **31.88** | **17.86** | **55.33** | **——** |
| *Carex parvula* | 783.49 | 35.41 | 25.47 | 30.91 | 18.60 | 54.43 | <https://doi.org/10.1038/s41598-022-08783-z> |
| *Carex kokanica* | 673.40 | 34.68 | 23.84 | 30.63 | 26.06 | 59.46 | <https://doi.org/10.1038/s41598-022-08783-z> |
| *Carex myosuroides* | 399.99 | 36.16 | 21.68 | 24.15 | 20.74 | 44.89 | <https://doi.org/10.1093/dnares/dsac049> |
| *Juncus inflexus* | 267.56 | 33.18 | 15.62 | 17.57 | 5.41 | 22.98 | <https://doi.org/10.1093/g3journal/jkac211> |
| *Juncus effusus* | 224.47 | 32.64 | 14.95 | 16.95 | 3.82 | 20.77 | <https://doi.org/10.1093/g3journal/jkac211> |
| *Bolboschoenus planiculmis* | 238.01 | 35.30 | 9.62 | 10.78 | 11.04 | 21.82 | https://doi.org/10.1093/gbe/evae039 |
| *Cyperus esculentus* | 225.60 | 35.60 | 8.67 | 10.50 | 5.02 | 34.14 | <https://doi.org/10.1093/gbe/evad027> |
| *Carex cristatella* | 301.64 | 33.86 | 6.86 | 8.23 | 9.97 | 18.20 | <https://doi.org/10.1093/g3journal/jkac211> |
| *Carex scopari* | 298.04 | 33.76 | 6.32 | 7.56 | 11.68 | 19.24 | <https://doi.org/10.1093/g3journal/jkac211> |
